# Supplementary figures and images for: Risk factors for dementia in the context of cardiovascular disease: A protocol of an overview of reviews
Source: PLoS One. 2022 Jul 21;17(7):e0271611. doi: 10.1371/journal.pone.0271611 (PMC9302739; doi:10.1371/journal.pone.0271611)

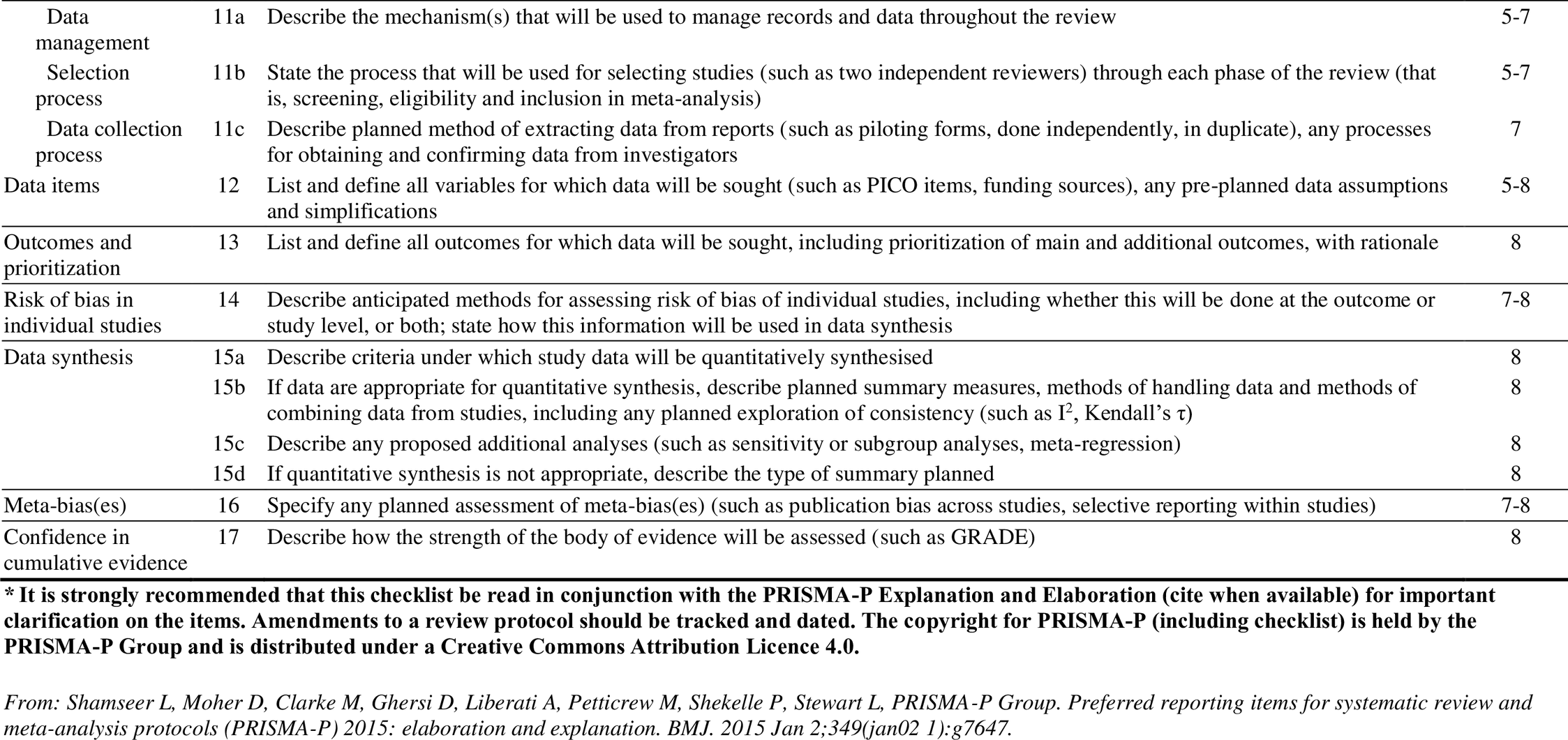

Supplement: S1 File — (ZIP) [file pone.0271611.s001.zip › S1 TIFF files/S1(2).tif]

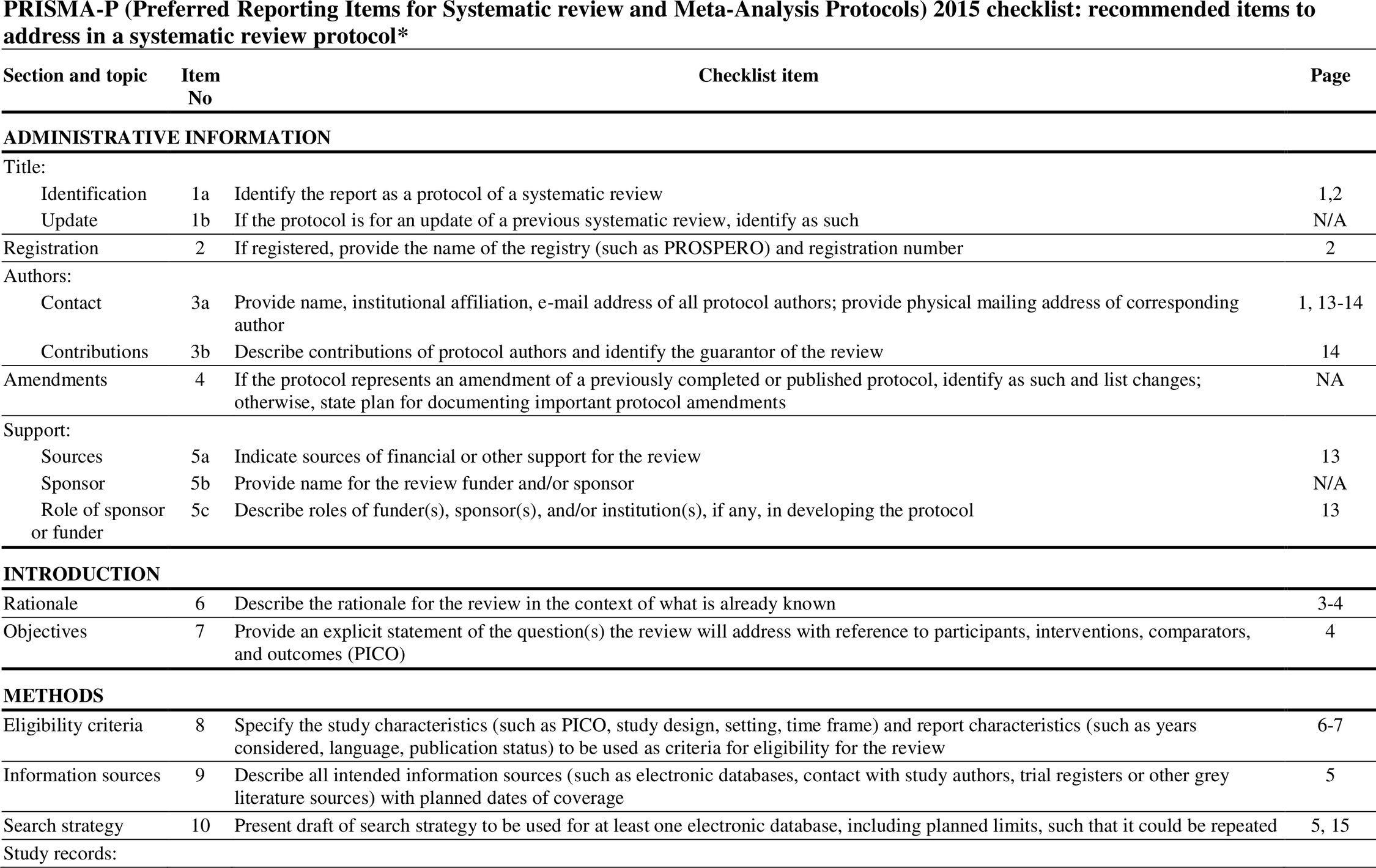

Supplement: S1 File — (ZIP) [file pone.0271611.s001.zip › S1 TIFF files/S1 (1).tif]

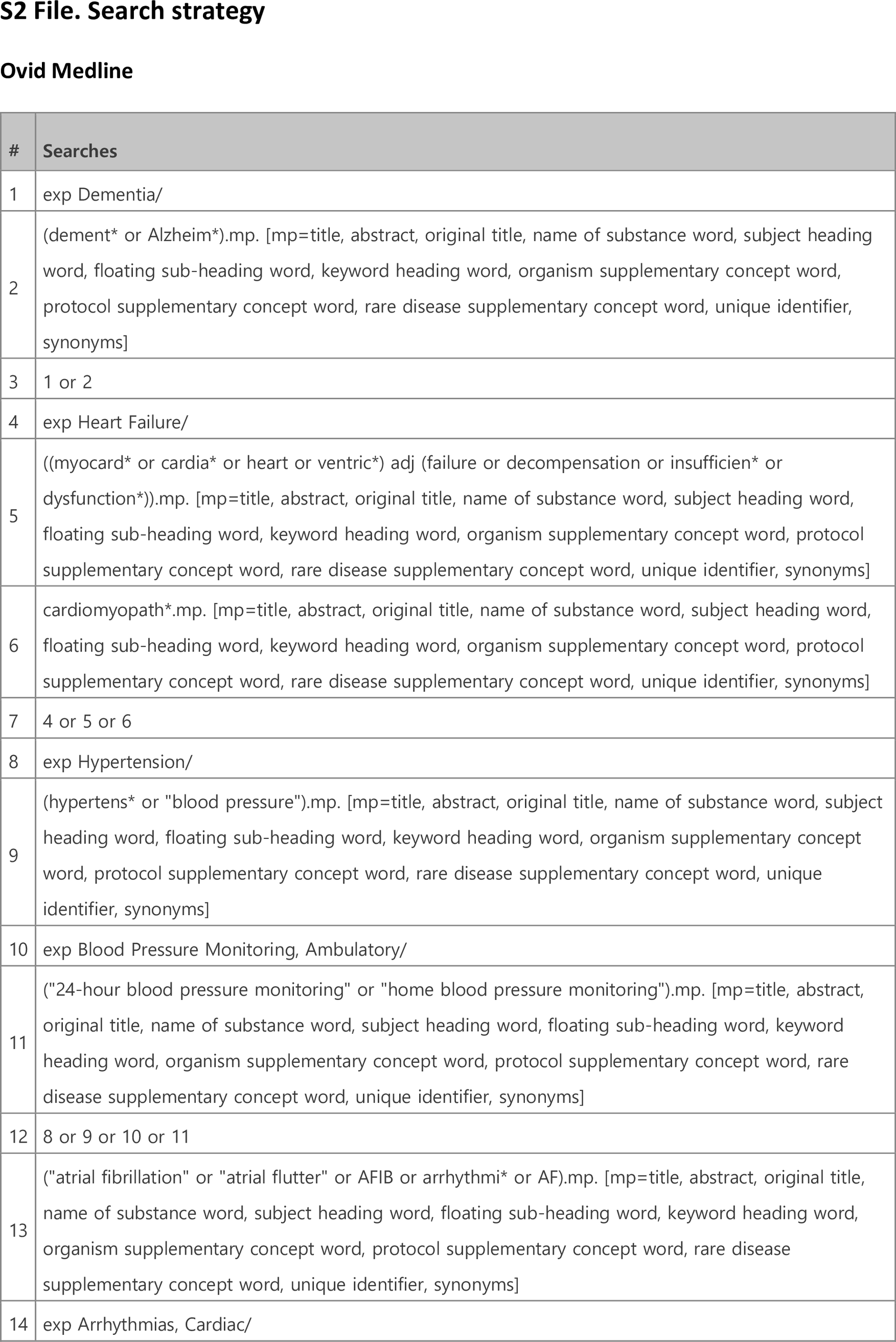

Supplement: S2 File — (ZIP) [file pone.0271611.s002.zip › s2 TIFF files/S2 file (1).tif]

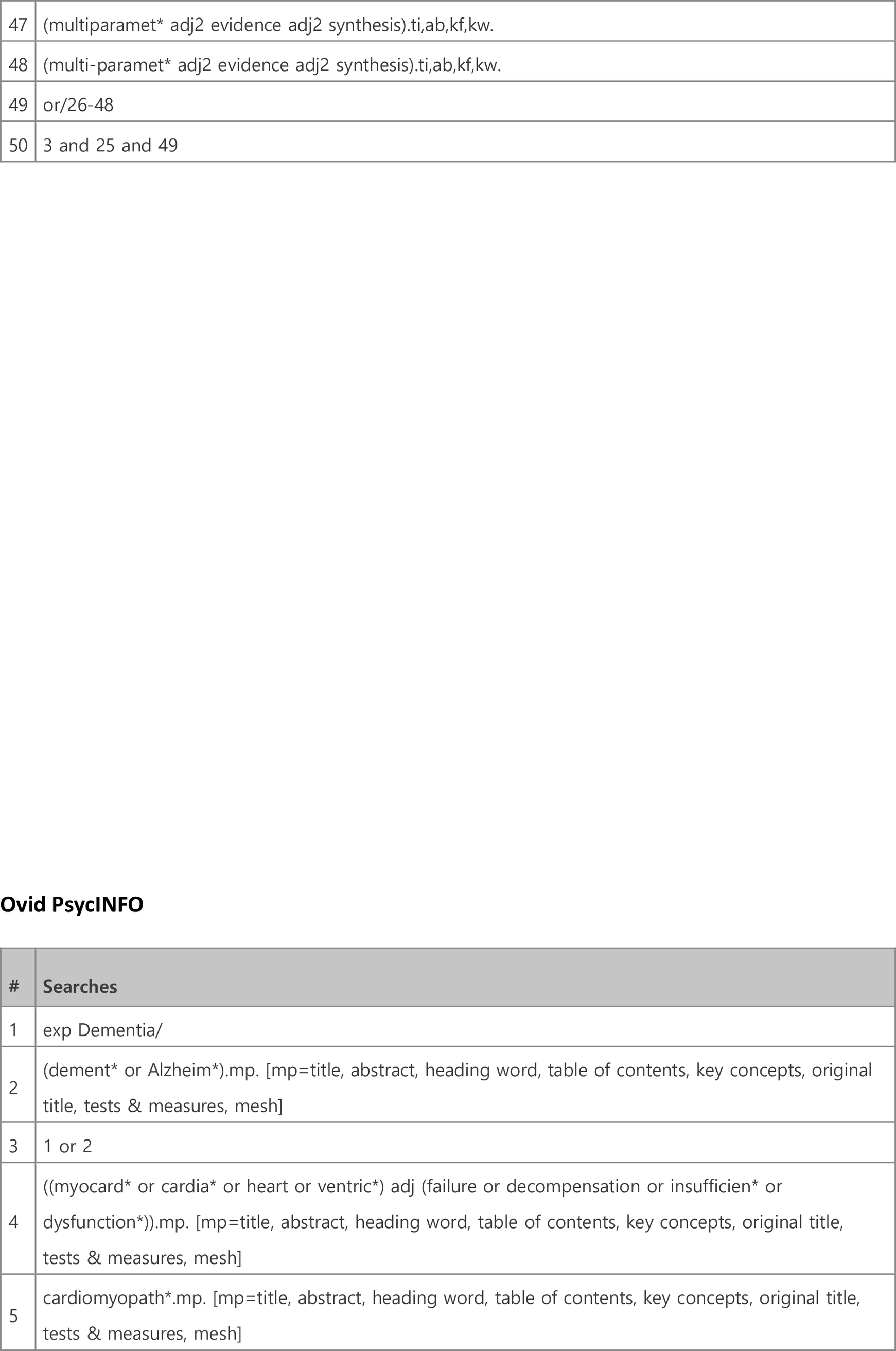

Supplement: S2 File — (ZIP) [file pone.0271611.s002.zip › s2 TIFF files/S2 file (6).tif]

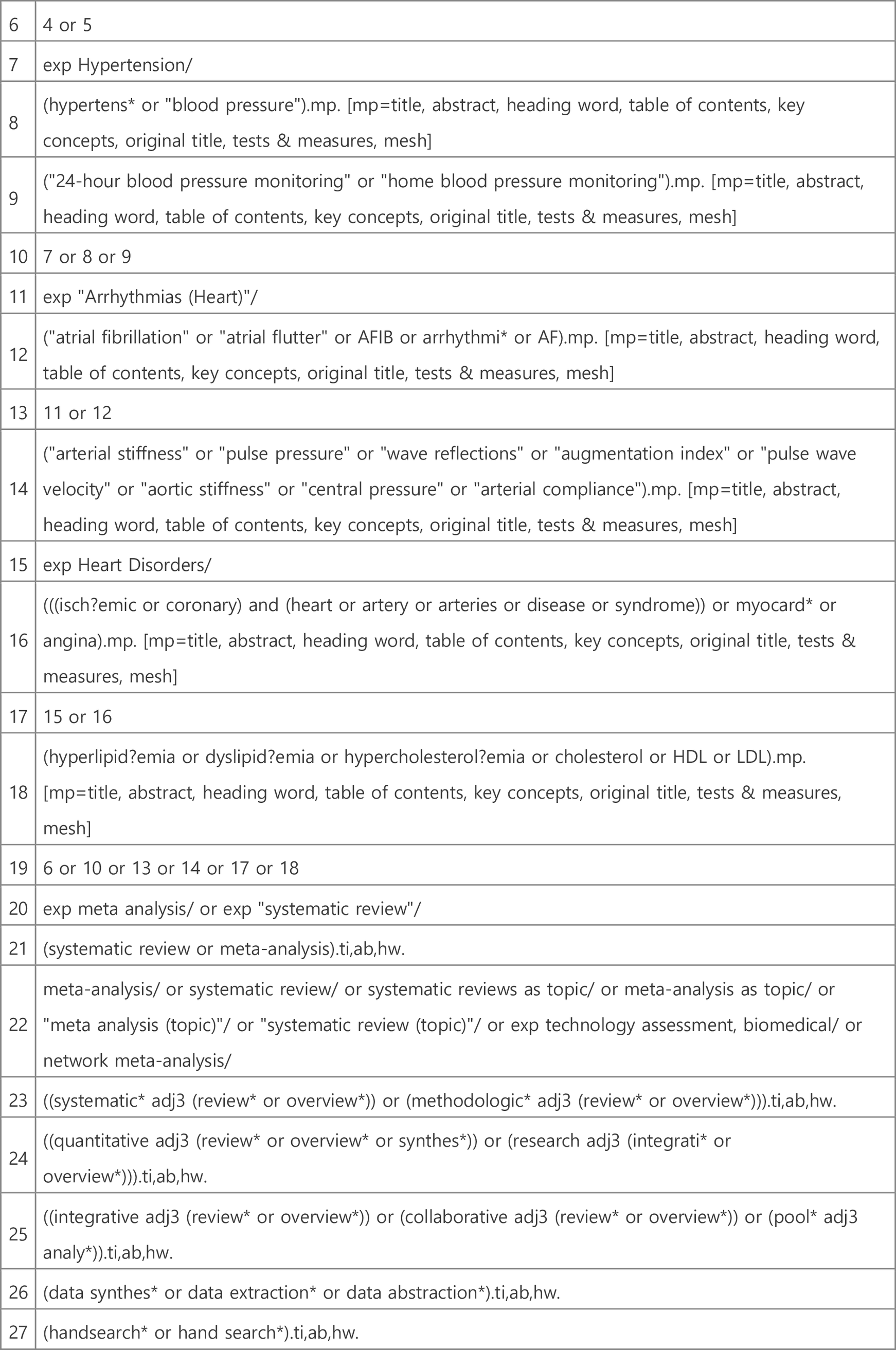

Supplement: S2 File — (ZIP) [file pone.0271611.s002.zip › s2 TIFF files/S2 file (7).tif]

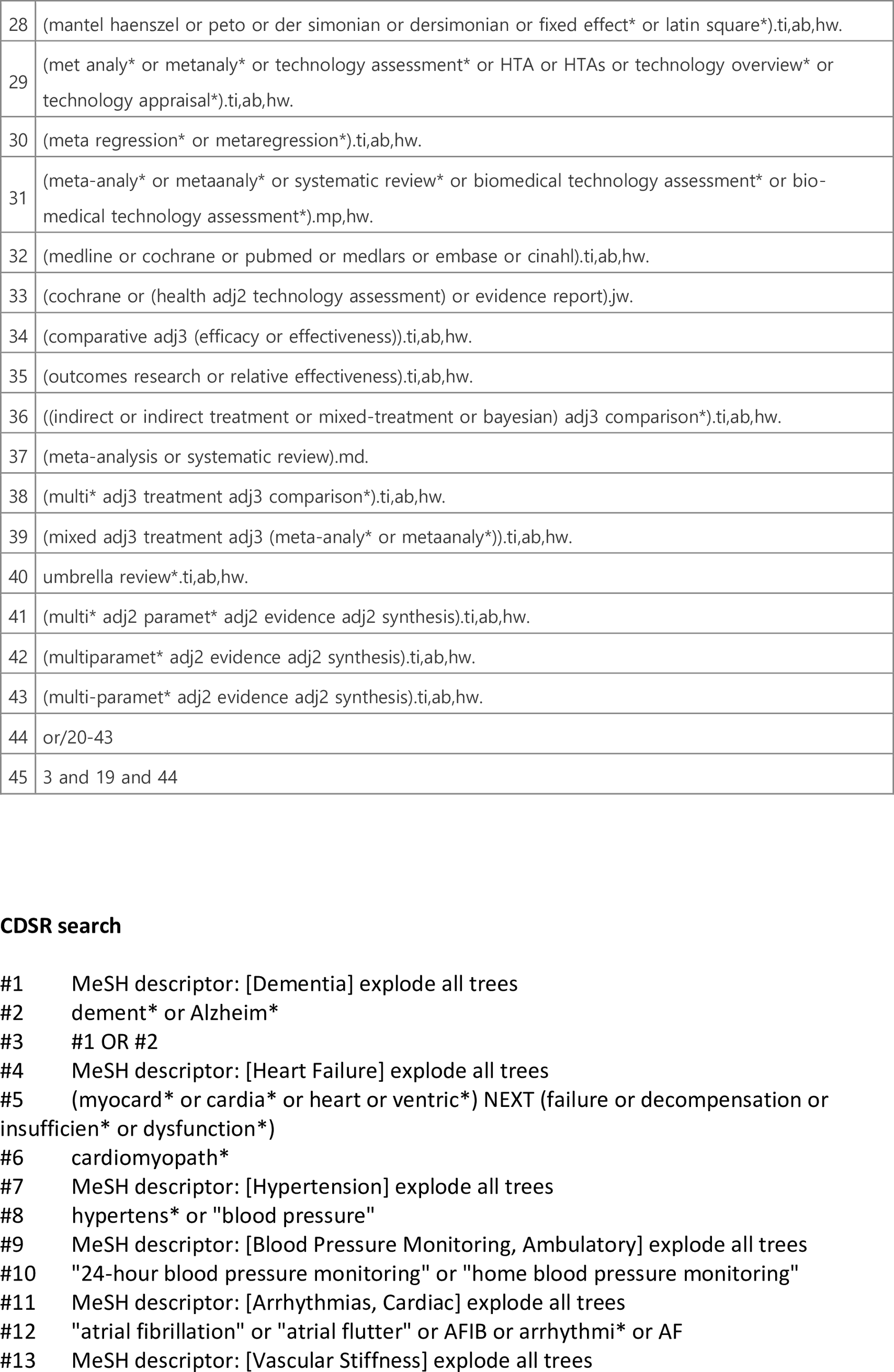

Supplement: S2 File — (ZIP) [file pone.0271611.s002.zip › s2 TIFF files/S2 file (8).tif]

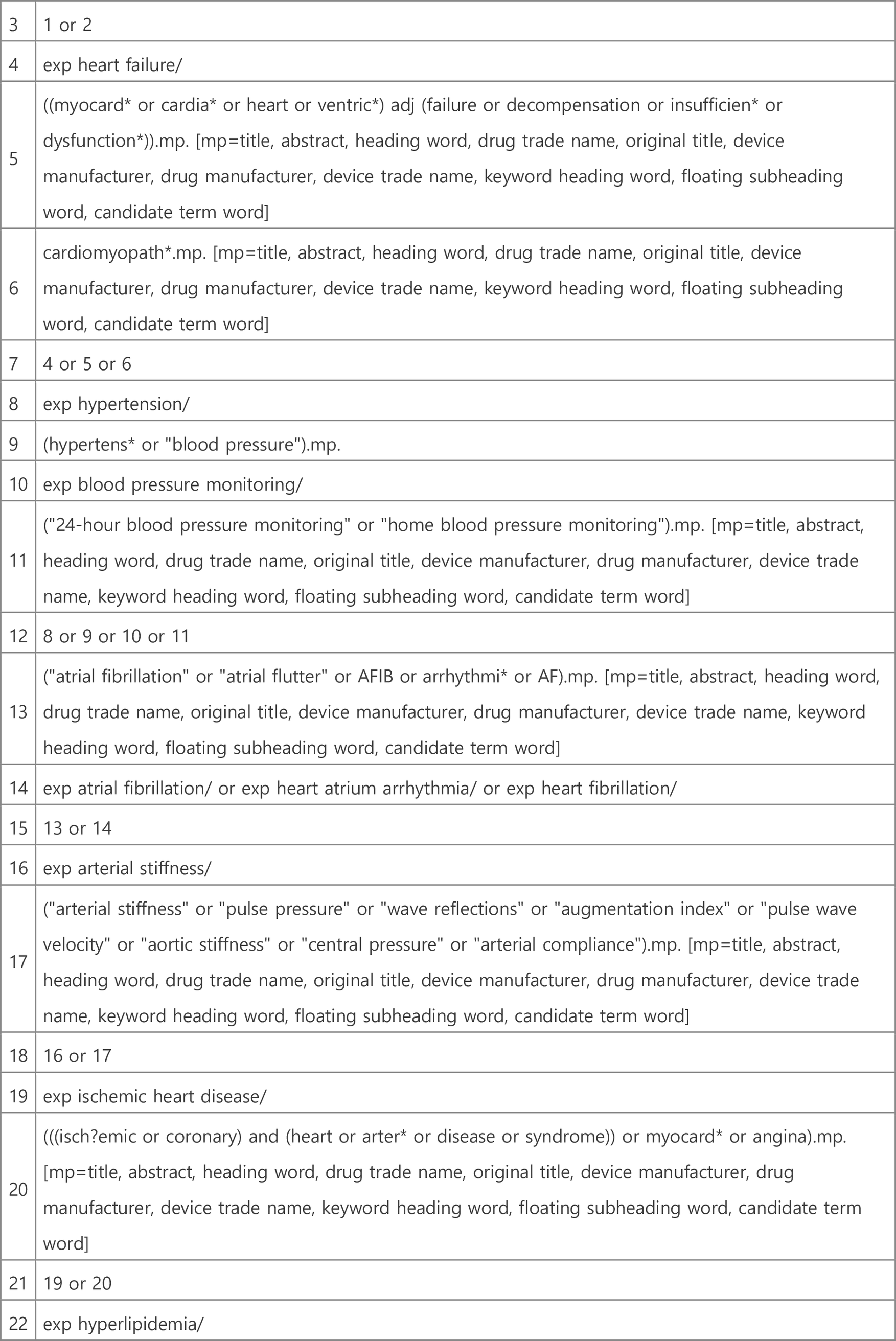

Supplement: S2 File — (ZIP) [file pone.0271611.s002.zip › s2 TIFF files/S2 file (4).tif]

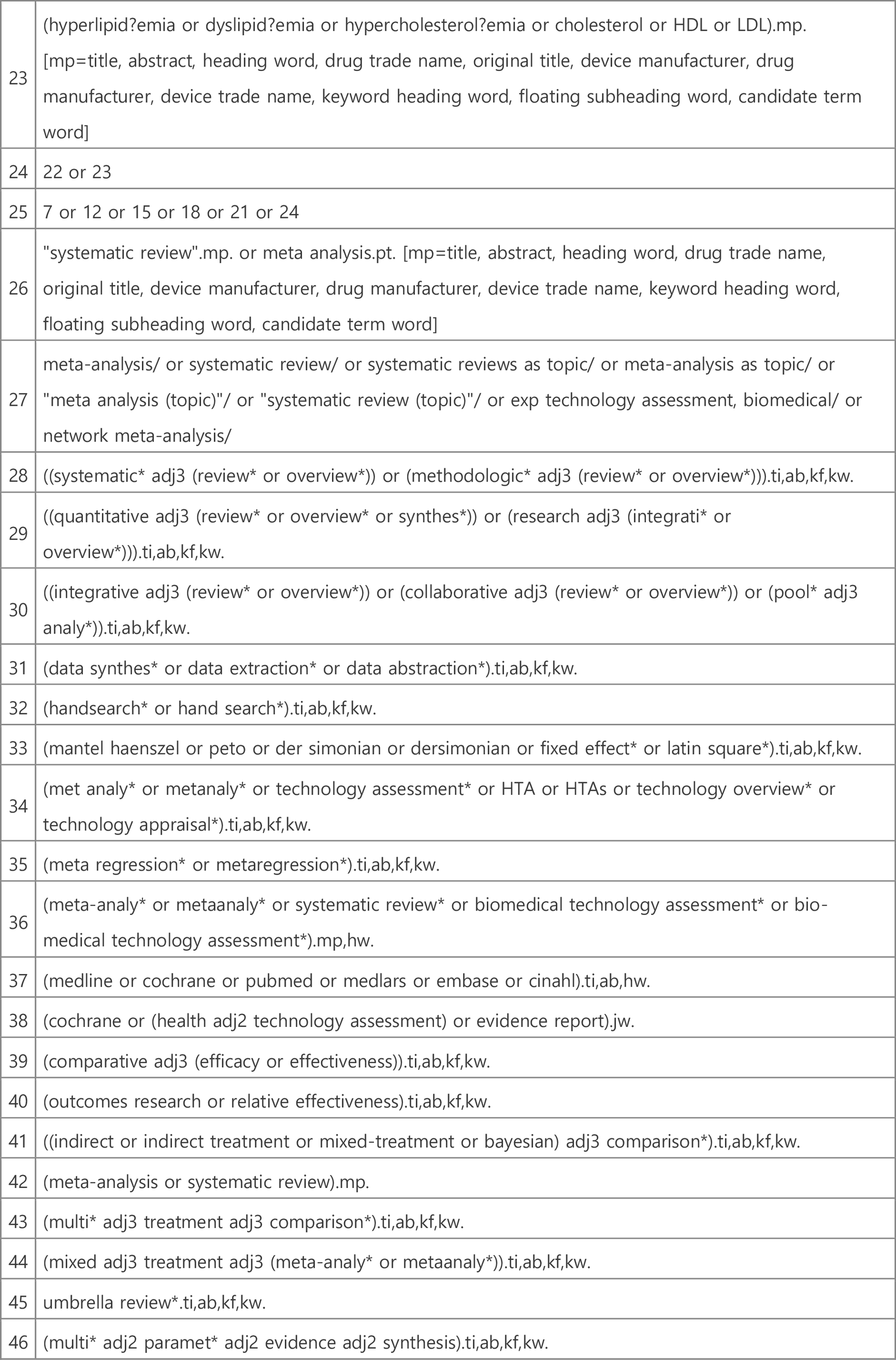

Supplement: S2 File — (ZIP) [file pone.0271611.s002.zip › s2 TIFF files/S2 file (5).tif]

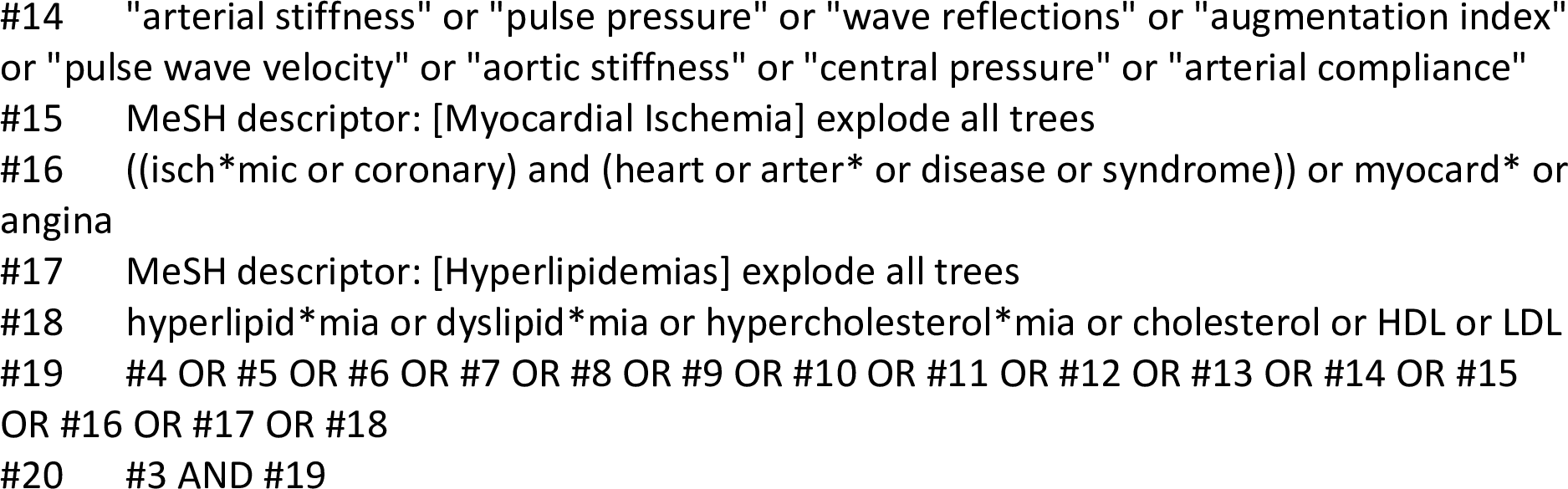

Supplement: S2 File — (ZIP) [file pone.0271611.s002.zip › s2 TIFF files/S2 file (9).tif]

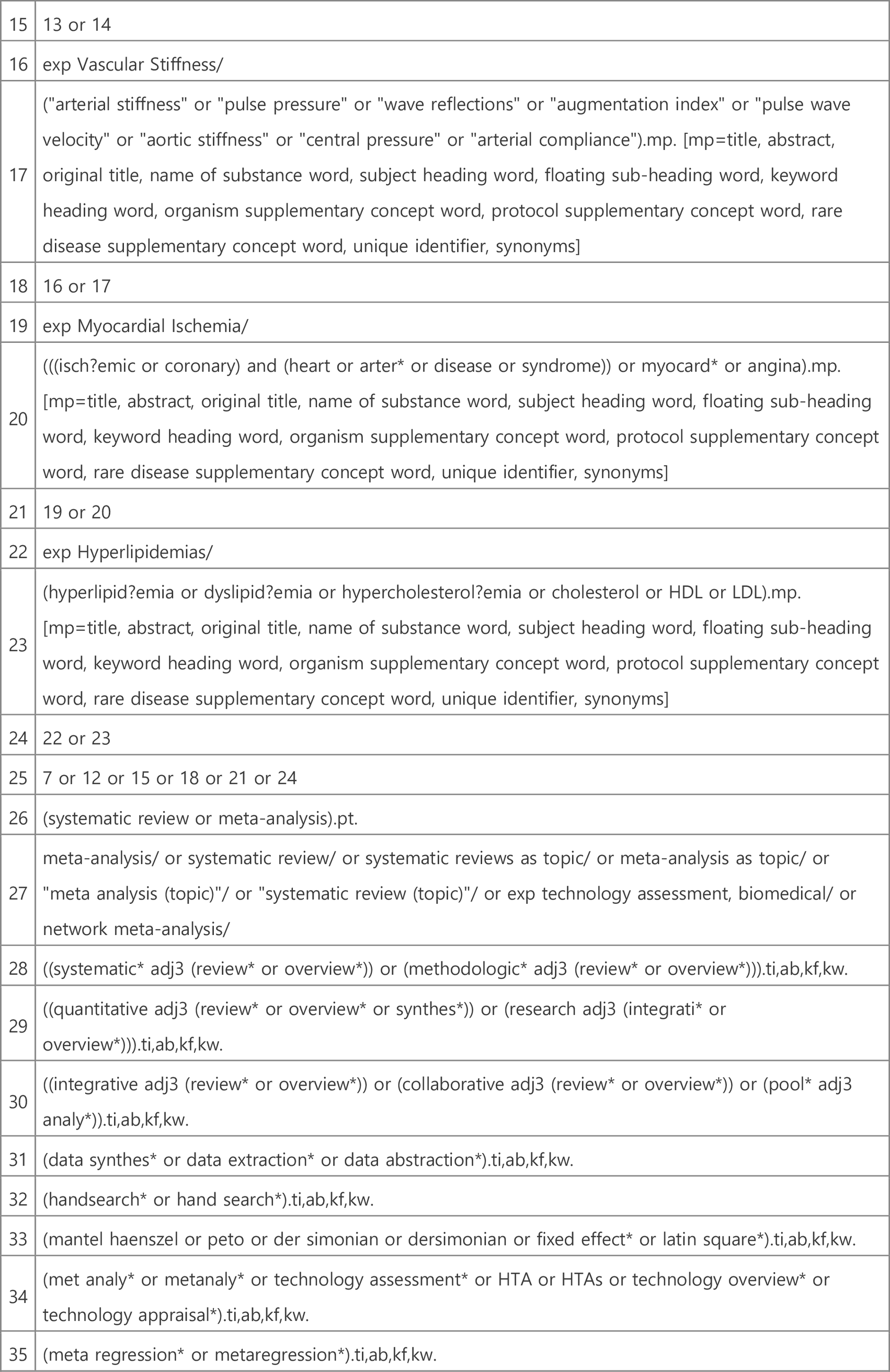

Supplement: S2 File — (ZIP) [file pone.0271611.s002.zip › s2 TIFF files/S2 file (2).tif]

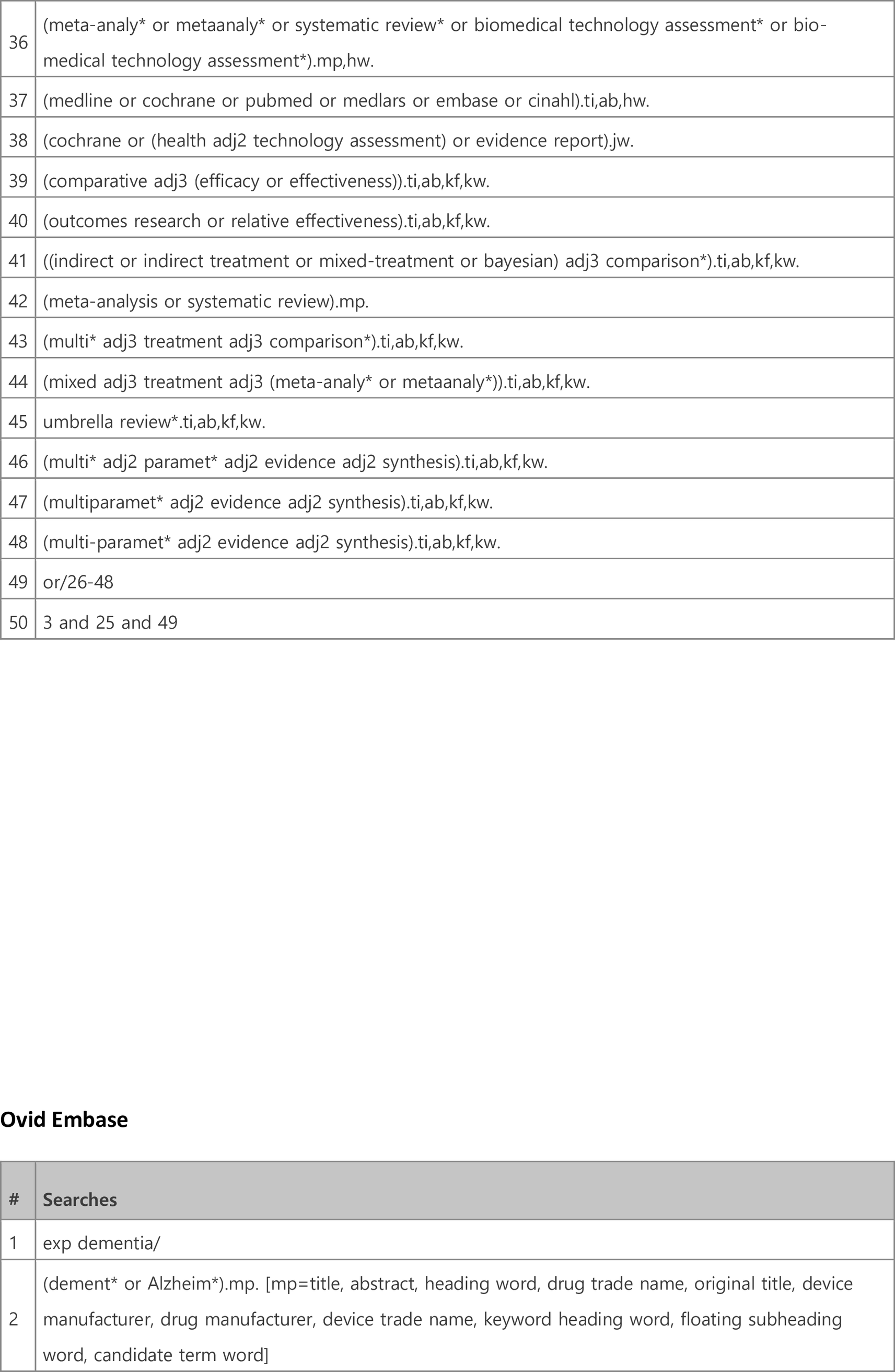

Supplement: S2 File — (ZIP) [file pone.0271611.s002.zip › s2 TIFF files/S2 file (3).tif]
