# Supplementary material for: Risk factors for dementia in the context of cardiovascular disease: A protocol of an overview of reviews
Source: PLoS One. 2022 Jul 21;17(7):e0271611. doi: 10.1371/journal.pone.0271611 (PMC9302739; doi:10.1371/journal.pone.0271611)
Supplement: Attachment — Submitted filename: Response to Reviewers.docx [file pone.0271611.s003.docx]

**Cover letter and reviewer comments**

Dear Professor Leist,

Thank you for the opportunity to revise and re-submit our manuscript “Risk factors for dementia in the context of cardiovascular disease: a protocol of an overview of reviews” [PONE-D-22-04170]. We would also like to thank the reviewers for providing extremely helpful feedback on the text. Our response to the comments and the changes we have made are attached below. All changes made in the manuscript are done in tracked changes.

All authors have approved the final version and there are no conflicts of interest to declare.

I look forward to hearing from you.

We would like to thank the reviewers for their extremely helpful comments. All the changes suggested have been made and are detailed below.

| **Reviewer #1** | | |
| --- | --- | --- |
| **Comment** | **Response** | **Location of change** |
| I would like to thank the authors for submitting this well-written protocol. | Thank you for your positive response to our paper and for all the feedback. | N/A |
| **Background** - This section could be better contextualized. For instance, the 2020 Lancet commission highlighted 12 risk factors for dementia prevention. Which could be identified as, or associated with cardiovascular risk factors? What is the approximate percentage reduction in dementia prevalence if these risk factors were eliminated in this context? | Thank you for providing the reference. We have added a discussion of the Lancet Commission findings and highlight the population attribute risk fractions for CVD (e.g., hypertension) and its risk factors including alcohol consumption, physical inactivity, smoking and obesity. | Lines 44-52  New Reference added |
| Are there any possible policies that can be developed with the findings of this systematic review? | Yes, identifying key risk factors for the development of dementia in the context of CVD will have important policy implications in terms of informing new campaigns to improve both heart and brain health. | Lines 78-80 |
| Briefly report whether gender, ethnicity, and other factors could influence the cardiovascular risk factors. | We have expanded the text on risk factors that could influence the association between CVD and dementia including age, sex, ethnicity and genetics | Lines 52-63 |
| Search strategy – In this section, it would be necessary to clearly state that MeSH terms will be used, and that search strategy will be adapted for each database. | We have added the specific MeSh terms to the Methods section. | Line 106, 110 |
| **Data synthesis** - How these results will be organized? Will the authors provide tables including sample characteristics, overall results, and other possible descriptions? | Yes, a results table will be provided that summaries each review paper. We have updated the “Data Synthesis” section to make this clear. | 167-184 |
| **Discussion** – As there are other risk factors associated with dementia, the authors should rephrase the following statement: “ Reducing the number of people with dementia.” | We have deleted the sentence to keep the text focused on CVD and dementia. |  |
| Finally, other systematic review references could be included:  Purnell, C., Gao, S., Callahan, C. M., & Hendrie, H. C. (2009). Cardiovascular risk factors and incident Alzheimer disease: a systematic review of the literature. Alzheimer disease and associated disorders, 23(1), 1–10. https://doi.org/10.1097/WAD.0b013e318187541c  Anstey, K. J., Lipnicki, D. M., & Low, L. F. (2008). Cholesterol as a risk factor for dementia and cognitive decline: a systematic review of prospective studies with meta-analysis. The American journal of geriatric psychiatry: official journal of the American Association for Geriatric Psychiatry, 16(5), 343–354. https://doi.org/10.1097/JGP.0b013e31816b72d4 | Thank you for the references; they have been added. |  |
| **Reviewer #2** | | |
| In this manuscript, the authors present the protocol for an overview of systematic reviews aimed to provide a synthesis of the evidence on the main dementia risk factors in people with a history of cardiovascular disease. The link between cardiovascular disease and dementia risks is a key question for dementia prevention. On this regard, this rigorously planned study aims to/will likely provide clarifications in the large body of evidence currently available, as well as important insights for future dementia-focused research. The manuscript is well written, and the study premises and design are clearly presented. I have only a few minor comments related to specific information to be provided ad their location in the manuscript. | Thank you for your positive feedback and for the comments which have improved the paper. | N/A |
| At the end of the methods section of the abstract different “cardiovascular groups” are mentioned (line 41-42). I assume that this refers to the different CVD diagnoses mentioned above (lines 33-34). However, the term cardiovascular group is fairly vague and used only in this instance. Another wording, e.g., “cardiovascular diagnoses” would more clearly explain what these groups include. | We have removed “cardiovascular group” and used the term “cardiovascular diagnoses” as suggested. | Line 24 |
| Please, state also in the abstract that the overview of systematic review will follow the PRISMA statement/guidelines | Added as requested. | Line 21-22 |
| In the “Quality assessment and evidence grading” section, it is not clear whether the result of the AMSTAR assessment will be used to further select studies (e.g., for inclusion/exclusion in the data synthesis). However, in the discussion section (lines 190-192) the potential low quality of the studies included is listed as one of the limitations, which suggest that this criterion will not be applied and low quality studies will be excluded only in sensitivity analysis. Regardless of whether studies will be selected based on the results of the AMSTAR assessment, the authors strategy on this regard should be clearly stated in the methods section and not only in the discussion. In the discussion, a clear explanation of the rationale whereby studies will be included regardless of their quality assessment, which means also studies with quality rated as “low”/”critically low” could be included in the data synthesis, should be provided. | We will not use the AMSTAR assessment to select studies for inclusion into the review. If a meta-analysis is possible, we will run a sensitivity analysis excluding studies rated as being of low quality/high risk of biased to determine their effects on the overall results. To make this clear we have updated the text in the sections titled “Quality assessment and evidence grading” and “Data synthesis”. | Line 152-159  Line 167-184 |
| - The section “Data Synthesis” lacks crucial details that should be prospectively provided in relation to the data synthesis methods and that are either missing from or presented in other sections (e.g., discussion) of the manuscript. In particular, these include: 1. specific statistics methods that will be applied to assess homogeneity/heterogeneity of the data to select studies for pooling, 2. criteria/cut-offs used to make this selection based on the results of the assessments mentioned in point 1, and 3. planned sensitivity analyses. | Thank you for the valuable comment. We have added specific details on the analyses that will be conducted. | Line 167-184 |
